# 5-year oncological outcomes in left-sided malignant colonic obstruction: stent as bridge to surgery

**DOI:** 10.1186/s12876-023-02903-3

**Published:** 2023-07-31

**Authors:** Noura S Alhassan, Sulaiman A AlShammari, Razan N AlRabah, Amirah M AlZahrani, Maha-Hamadien Abdulla, Thamer A Bin Traiki, Ahmad M Zubaidi, Omar A Al-Obeed, Khayal A Alkhayal

**Affiliations:** 1 Colorectal Research Chair, Department of Surgery, College of Medicine, King Khalid University Hospital, King Saud University, Riyadh, Saudi Arabia; 2 College of Medicine, King Saud University, Riyadh, Saudi Arabia

**Keywords:** SEMS, Oncological outcomes, Malignant colonic obstruction, Upfront surgery

## Abstract

**Background:**

A considerable number of patients with colon cancer present with a colonic obstruction. The use of self-expanding metallic stents (SEMS) as a bridge to surgery (BTS) in potential curative patients with left-sided colonic cancer obstruction remains debatable. Therefore, this study aimed to investigate the 5-year oncological outcomes of using a SEMS as a BTS.

**Methods:**

All patients with left-sided malignant colon obstruction who underwent curative surgery with no metastasis upon presentation between March 2009 and May 2013 were retrospectively reviewed and analyzed.

**Results:**

A total of 45 patients were included, 28 patients underwent upfront surgery, and 17 patients had a stent as a bridge to surgery. T4 stage was statistically significantly higher in patients who had a SEMS as a BTS (35.3% vs. 10.7%) *(p-value 0.043)*. The mean duration in days of the SEMS to surgery was 13.76 (SD 10.08). TNM stage 3 was a prognostic factor toward distant metastasis (HR 5.05). When comparing patients who had upfront surgery to those who had a SEMS as a BTS, higher 5-year disease-free survival (75% vs. 72%) and 5-year overall survival (89% vs. 82%) were seen in patients who had upfront surgery. However, both were statistically insignificant.

**Conclusion:**

Using self-expanding metallic stents as a bridge to surgery yields comparable 5-year survival and disease-free survival rates to upfront emergency surgery. The decision to use SEMS versus opting for emergency surgery should be made after careful patient selection and with the assistance of experienced endoscopists.

**Trial registration:**

N/A.

## Background

Colorectal cancer (CRC) is a significant cause of morbidity and mortality worldwide. It is the third most common cancer and the second most common cause of death globally [1]. The incidence of CRC cancer in Saudi Arabia has increased significantly in the last two decades [2].

13% of patients with CRC present initially with an acute large bowel obstruction [3]. Emergency upfront surgery was the sole intervention for those patients to relieve the obstruction despite its high risk of mortality and morbidity compared to elective surgery due to patients’ poor conditions related to age, malnutrition, sepsis, and inadequate bowel preparation [4, 5]. However, self-expanding metallic stents (SEMS) as a bridge to surgery (BTS) are gaining popularity as an alternative option for colonic decompression prior to surgery [6].

The success rate of endoscopic stenting is reported to be up to 90% [7]. SEMS relieves colonic obstruction, turning an emergency operation into an elective resection, which has a higher primary anastomosis rate, limiting postoperative complications and reducing the rate of stoma creation [8]. Many studies documented a comparable oncological outcome between SEMS as BTS to upfront emergency surgery [9, 10]. In addition, it allows optimization of the patient’s condition and proper tumor staging before definitive intervention [11]. Several authors do not recommend using SEMS as some reports found it to be associated with tumor cell dissemination, tumor perforation, and increased local recurrence rates [12–17].

The long-term oncological outcomes of using SEMS as a BTS in potential curative patients with left-sided colonic cancer obstruction remain debatable. We have previously studied the short-term outcomes and complications following SEMS placement in malignant colonic obstruction at our center [18]. This study aimed to investigate the 5-year oncological outcomes of using SEMS as a BTS.

## Methodology

After the approval of the Ethical Institutional Review Board (IRB) at King Saud University. We retrospectively reviewed the medical records of all patients with left-sided malignant colon obstruction at King Saud University Medical City (KSUMC), an academic medical institution and a tertiary hospital with 1200 beds in Riyadh, Saudi Arabia.

Patients with left-sided non-metastatic malignant colon obstruction who underwent curative surgery upon presentation between March 2009 and May 2013 were included in this study. Patients managed palliatively, less than 18 years, right-sided colon obstruction, rectal cancer, and benign etiology were excluded. Patients were divided into two groups; those who had an emergency upfront surgery and those who had a SEMS as a BTS.

### Management pathway

All patients were assessed and treated initially by the general surgery on-call or acute care team, and the colorectal consultant on-call was involved in the management if needed. The decision of SEMS placement versus upfront surgery was discussed with the gastroenterologist on-call. Patients with hemodynamic instability or generalized peritonitis were not fit for SEMS placement. The following working day, the colorectal team reviews and manages all the cases, in addition to presenting all cases at the tumor board meeting for further management.

### The procedure for SEMS placement

For all patients, the endoscopic placement of SEMS was done by senior gastroenterologists. Each patient was evaluated clinically and radiologically to assess the lesion. The colon was prepared with a water-soluble enema prior to the procedure. Uncovered (WallFlex) colonic stents were used, 22 mm in diameter and 60 or 90 mm in length. The stents were placed endoscopically under fluoroscopic guidance. If the scope did not pass through the obstruction, a 0.035-inch guide wire was used to help with stent placement. The patients were monitored prior to, during, and after the procedure. Any adverse events were recorded and addressed.

### Statistical analysis

Data were analyzed using Statistical Package for Social Studies (SPSS 22; IBM Corp., New York, NY, USA). Continuous variables were expressed as mean ± standard deviation and median, and Categorical variables were expressed as percentages. The t-test was used for continuous variables with normal distribution, and Mann-Whitney Test was used for continuous variables without normal distribution. The chi-square test and Fisher exact test were used for categorical variables. Cox proportional hazards regression was performed to estimate hazard ratios (HR) (95% CI). Survival curves were estimated by the Kaplan–Meier method. A *p-value < 0.05* was considered statistically significant.

## Results

Between March 2009 and May 2013, 45 patients with left-sided malignant colon obstruction underwent colonic resection. Of these, 28 patients (62.2%) had an upfront surgery, and 17 (37.8%) had a SEMS as a BTS. Following stent placement, only one patient had an obstruction with impacted stool, and was managed with colonoscopy-mediated irrigation. Total colectomy was done for two patients, one in each group, due to either questionable blood supply or multiple serosal tears in the distended right colon. Worth to mention the total colectomy performed in the SEMS group was for the same patient who had complicated stent stool impaction. The baseline characteristics of both groups are illustrated in Table 1.

**Table 1:** Baseline characteristics (n = 45)

		SEMS (N = 17)		Upfront surgery (N = 28)		P value
		Number	%	Number	%	
Age^#^		59.59	11.80	54.29	12.63	0.181
Gender	Male	11	64.71	9	32.14	0.033*
	Female	6	35.29	19	67.86	
Co-morbidities	Diabetes Mellitus	7	41.18	7	25.00	0.256
	Hypertension	6	35.29	10	35.71	0.977
	Bronchial Asthma	2	11.76	3	10.71	0.635
	Hypothyroidism	2	11.76	2	7.14	0.489
	Stroke	2	11.76	28	100.00	0.137
	IHD/HF	3	17.65	2	7.14	0.270
	Dyslipidemia	1	5.88	2	7.14	0.684
Cancer Site	Splenic Flexure	1	5.88	1	3.57	0.388
	Descending colon	3	17.65	6	21.43	
	Sigmoid	13	76.47	17	60.71	
	Rectosigmoid	0	0.00	4	14.29	
TNM stage	Stage 1	0	0.00	3	10.71	0.231
	Stage 2	5	29.41	12	42.86	0.367
	Stage 3	12	70.59	13	46.43	0.114
Approach of surgery	Laparoscopic	14	82.35	24	85.71	0.538
	Converted to open	3	17.65	1	3.57	0.144
	Open	3	17.65	4	14.29	0.538
Type of surgery	Left hemicolectomy	4	23.53	10	35.71	0.304
	Total colectomy	1	5.88	1	3.57	0.618
	Anterior resection	12	70.59	17	60.71	0.366

* Significant *p-value*, ^*#*^ Mean, SD

IHD/HF: Ischemic Heart disease/Heart failure

Clinical tumor parameters are presented in Table 2. The T2 stage was found only in patients who had upfront surgery; T3 was similar between both groups, whereas the T4 stage was significantly higher in patients who had a SEMS as a BTS (35.3% vs. 10.7%) *(p-value 0.043)*. The mean duration in days of SEMS to curative surgery was 13.76 (± 10.08).

**Table 2:** Clinical tumor petameters (n = 45)

		SEMS (N = 17)		Upfront surgery (N = 28)		P value
		Number	%	Number	%	
T stage	T2	0	0	5	17.9	0.043*
	T3	11	64.7	20	71.4	
	T4	6	35.3	3	10.7	
Lymph node dissected^#^		19.41	6.14	19.61	8.25	0.925
< 12 lymph nodes dissected		1	5.9	2	7.1	0.684
Positive Lymph node		12	70.6	13	46.4	0.114
Perineural invasion		3	17.6	4	14.3	0.538
Lymphovascular invasion		2	11.8	5	17.9	0.462
Mucin production		2	11.8	2	7.1	0.489
Adjuvant chemotherapy		15	88.2	26	92.9	0.489

* Significant *p-value*, ^*#*^ Mean, SD

**Fig. 1:**
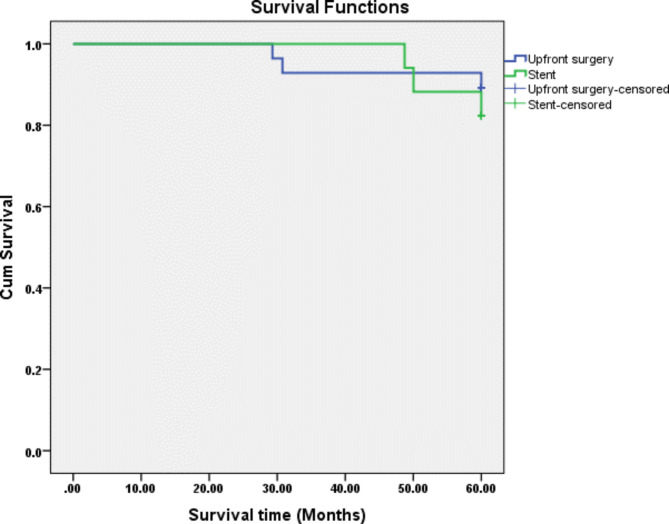
Cumulative Kaplan– Meier survival estimates for Upfront surgery and SEMS

**Fig. 2:**
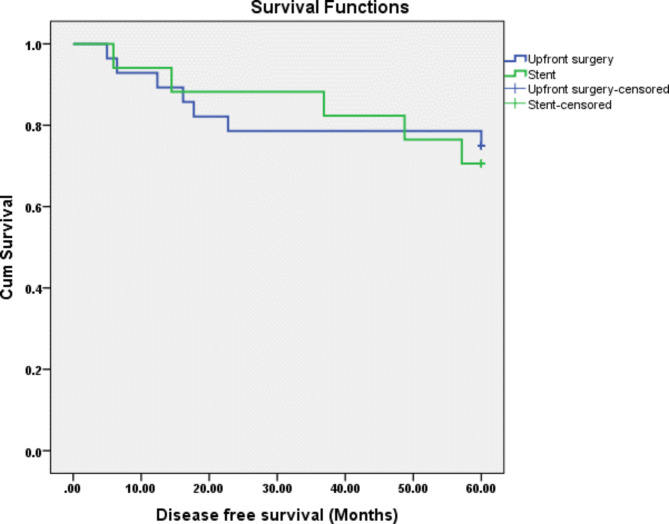
Cumulative Kaplan–Meier Disease free survival curve for Upfront surgery and SEMS

Oncological outcomes are summarized in Table 3. The overall local and distant recurrence rates were 4.44% and 26.67%, respectively. Local recurrence was seen only in the SEMS group, with one patient having a recurrence at the anastomosis site and one at the surgical bed. The overall and disease-free survival rates were 87% and 72%, respectively. When comparing patients who had upfront surgery to those who had a SEMS as a BTS, higher 5-year disease-free survival (75% vs. 71%) and 5-year overall survival (89% vs. 82%) were seen in patients who had upfront surgery. However, both were statistically insignificant, as seen in Figs. 1 and 2.

**Table 3:** Oncological outcomes (n = 45)

		SEMS (N = 17)		Upfront surgery (N = 28)		P value
		Number	%	Number	%	
Local recurrence		2	11.8	0	0	0.137
Distant metastasis		5	29.4	7	25.0	0.746
Site of Distant metastasis	Liver	2	11.8	4	14.3	0.593
	Lung	2	11.8	5	17.9	0.462
	Peritoneal	0	0	1	3.6	0.622
	Ovaries	0	0	1	3.6	0.622
	Brain	0	0	1	3.6	0.622
Surgery to distant metastasis duration in months^#^		37.16	22.44	21.64	22.66	0.412
Surgery to mortality duration in months^#^		52.99	6.28	47.44	30.17	0.700
Overall survival in months^##^		58.75	(56.76–60.75)	57.86	(54.36–61.36)	0.538
Disease-free survival in months^##^		51.95	(44.15–59.74)	50.02	(42.28–57.75)	0.803

^*#*^ Mean, SD, ^##^Mean, 95% CI

Prognostic factors toward distant metastasis are shown in Table 4. TNM stage 3 was a risk factor for distant metastasis *(p-value 0.025).* The univariate regression model tested the significant factors, as shown in Table 5.

**Table 4:** Prognostic factors toward distant metastasis (n = 45)

		Distant metastasis				P value
		Yes (N = 12)		No (N = 33)		
		Number	%	Number	%	
Age^#^		54.67	12.12	56.88	12.71	0.568
Gender	Male	5	41.67	15	45.45	0.821
	Female	7	58.33	18	54.55	
Cancer Site	Splenic Flexure	1	8.33	1	3.03	0.578
	Descending colon	2	16.67	7	21.21	
	Sigmoid	7	58.33	23	69.70	
	Rectosigmoid	2	16.67	2	6.06	
Upfront surgery		7	58.33	21	63.64	0.764
SEMS		5	41.67	12	36.36	
T stage	2	1	8.33	4	12.12	0.864
	3	9	75.00	22	66.67	
	4	2	16.67	7	21.21	
TNM stage	Stage 1	0	0.00	3	9.09	0.384
	Stage 2	2	16.67	15	45.45	0.076
	Stage 3	10	83.33	15	45.45	0.025*
Positive lymph node		10	83.33	15	45.45	0.025*
< 12 lymph node dissected		1	8.33	2	6.06	0.616
Positive Lymph node^#^		2.25	3.05	1.33	2.33	0.109
Lymph node dissected^#^		21.42	6.93	18.85	7.61	0.122
Perineural invasion		4	33.33	3	9.09	0.069
Lymphovascular invasion		3	25.00	4	12.12	0.268
Mucin production		1	8.33	3	9.09	0.714
SEMS to surgery in days^#^		11.40	7.27	14.75	11.19	0.879
SEMS to surgery > 14 days		2	16.67	4	12.12	0.605
Adjuvant chemotherapy		11	91.67	30	90.91	0.714
Approach of surgery	Laparoscopic	9	75.00	29	87.88	0.268
	Converted to open	1	8.33	3	9.09	0.714
	Open	3	25.00	4	12.12	0.268
Type of surgery	Total colectomy	1	8.33	1	3.03	0.467
	Left hemicolectomy	4	33.33	10	30.30	0.558
	Anterior resection	7	58.33	22	66.67	0.606

* Significant *p-value*, ^*#*^ Mean, SD

**Table 5:** Cox proportional-hazards analysis

Variable		Univariate analysis		
		Hazard ratio	95% CI	P value
Prognostic factors toward distant metastasis				
TNM Stage 3		5.05	(1.10-23.09)	0.037*
**Prognostic factors toward survival**				
TNM stage	Stage 2	0.02	(0.00-18.78)	0.267
	Stage 3	58.24	(0.08–42,206)	0.236
Number of positive lymph nodes^#^		1.23	(1.01–1.51)	0.043*
Distant metastasis		669.68	(0.01-45936524)	0.252

* Significant *p-value*, ^*#*^ Mean, SD

Prognostic factors toward survival are displayed in Table 6. The significant factors were tested in the univariate regression model, as shown in Table 5. Survival rate correlated significantly with TNM stages 2 and 3 and distant metastasis. However, all of the factors were insignificant in the multivariant analysis.

**Table 6:** Prognostic factors toward survival (n = 45)

		Alive (N = 39)		Death (N = 6)		P value
		Number	%	Number	%	
Age^#^		56.51	12.04	54.83	16.14	0.832
Gender	Male	18	46.15	2	33.33	0.447
	Female	21	53.85	4	66.67	
Cancer Site	Splenic Flexure	2	5.13	0	0.00	0.152
	Descending colon	8	20.51	1	16.67	
	Sigmoid	27	69.23	3	50.00	
	Rectosigmoid	2	5.13	2	33.33	
Upfront surgery		25	64.10	3	50.00	0.407
SEMS		14	35.90	3	50.00	
T stage	2	5	12.82	0	0.00	0.601
	3	26	66.67	5	83.33	
	4	8	20.51	1	16.67	
TNM stage	Stage 1	3	7.69	0	0.00	0.644
	Stage 2	17	43.59	0	0.00	0.046*
	Stage 3	19	48.72	6	100.00	0.022*
Positive LN		19	48.72	6	100.00	0.022*
< 12 lymph node dissected		3	7.69	6	100.00	0.644
Positive Lymph node^#^		1.28	2.16	3.50	3.99	0.045*
Lymph node dissected^#^		19.13	7.83	22.17	3.60	0.150
Perineural invasion		5	12.82	2	33.33	0.320
Lymphovascular invasion		5	12.82	2	33.33	0.320
Mucin production		4	10.26	0	0.00	0.552
SEMS to surgery in days^#^		13.86	10.84	13.33	7.02	0.768
SEMS to surgery in > 14 days		5	12.82	1	16.67	0.728
Adjuvant chemotherapy	Yes	35	89.74	6	100.00	0.552
Approach of surgery	Laparoscopic	33	84.62	5	83.33	0.661
	Converted to open	4	10.26	0	0.00	0.552
	Open	6	15.38	1	16.67	0.661
Type of surgery	Total colectomy	1	2.56	1	16.67	0.252
	Left hemicolectomy	13	33.33	1	16.67	0.382
	Anterior resection	25	64.10	4	66.67	0.642
Distant metastasis		6	15.38	6	100.00	< 0.001*

* Significant *p-value*, ^*#*^ Mean, SD, ^*##*^ Median

## Discussion

The use of SEMS in malignant colonic obstruction has gained attention as an alternative decompression intervention prior to curative resection. In many studies, SEMS placement was associated with high clinical success and good short-term outcomes [7–10]. In spite of this, long-term oncological outcomes remain uncertain. This study presents the 5-year oncological outcomes of using a SEMS as a BTS in left-sided colonic cancer obstruction.

SEMS is a challenging procedure that requires an experienced gastroenterologist as it has a risk of perforation and failed decompression; the placement also depends on the site and length of tumor stenosis [19]. In the literature, some authors reported concerns regarding SEMS, which include a high risk of tumor cell dissemination, distant metastasis, and recurrence [16, 20]. In addition, it has been hypothesized that SEMS induces perineural invasion due to the expanding pressure pushing the obstruction onto the lumen wall, which causes cancer cell invasion to the surrounding vessels and tissues [20]. In this study, the perineural invasion was similar between both groups and lymphovascular invasion was (11.8% vs. 17.9%) in SEMS and upfront surgery, respectively.

A bridging interval between 9 and 14 days is ideal due to concerns about stent-related peritumor inflammation and adhesions, which may increase the technical difficulty of resection [6]. Moreover, a significantly increased risk of disease recurrence in patients with a bridging interval of more than two weeks has been reported [6]. In this study, the mean duration of days between SEMS to surgery was 13.76 (SD 10.08), and (64.7%) of patients had surgery in 14 days or less. We found that the distant recurrence rate was comparable in both groups, with no increased risk in patients with more than 14 days of SEMS to surgery interval *(p-value = 0.605).*

There have been mixed reports in regard to disease recurrence in the SEMS group compared to upfront surgery. Some studies reported higher recurrence rates in the SEMS group [10, 16, 17]; others reported no differences in local and distant recurrence rates [15, 21]. Our data showed that local recurrence was seen in only 2 patients (11.8%) in the SEMS group, and distant metastasis was seen in 5 (29.4%) vs. 7 (25%) in SEMS and upfront surgery groups, respectively. In addition, positive nodes, male sex, anastomotic dehiscence, and diffuse peritonitis were reported as prognostic factors toward recurrence [22]. The only risk factor for distant metastasis in our patient population was TNM stage 3 (HR 5.05).

Sabbagh et al. reported that the 5-year overall survival rate for left-sided colonic cancer was lower in the SEMS group than in the upfront surgery group (25% vs. 62%) *(p-value = 0.003)*, and the 5-year cancer-specific mortality rate was higher in the SEMS group (48% vs. 21%) *(p-value = 0.02)* [15]. However, no significant differences between the two groups in 5-year disease-free survival were found [15]. They included TNM stage 4, which was significantly higher in the SEMS group. The increased mortality observed in the SEMS group was likely due to the more advanced stage of the disease. A recent meta-analysis of randomized clinical trials showed no differences in recurrence and 3-year survival rate between the two groups [10]. Our current study only included patients with left-sided colonic obstruction and excluded patients who initially presented with distant metastasis. The 5-year overall survival and disease-free survival for patients who had upfront surgery compared to those who had a SEMS as a BTS were (89% vs. 82%) and (75% vs. 71%), respectively. However, both were statistically insignificant.

This study has a few limitations that should be considered. First, it is a retrospective cohort study, where inherent bias may be present. Second, the sample size is small, which could contribute to the statistical insignificance of different variables. Lastly, a standardized grading system for the severity of the obstruction is lacking, so implementing a scoring system would help categorize the patients appropriately. A prospective, multicentric study with a grading score for the obstruction severity, such as ColoRectal Obstruction Scoring System (CROSS), is recommended to assess the long-term oncological outcome [23].

## Conclusion

SEMS as a BTS has emerged as a viable option for patients with left-side colonic cancer obstruction. Our studies have shown that SEMS as a bridge to surgery is associated with comparable outcomes to upfront surgery. However, the optimal management strategy for these patients remains controversial. Therefore, tailored therapy for each patient presenting with left-side colonic cancer obstruction is advised.

## Data Availability

The datasets used and/or analyzed during the current study are available from the corresponding author upon reasonable request.

## List of abbreviations

SEMS: Self-expanding metallic stents
BTS: Bridge to surgery
CRC: Colorectal cancer
IRB: Institutional Review Board
KSUMC: King Saud University Medical City
